# *KLK6* and *KLK13* predict tumor recurrence in epithelial ovarian carcinoma

**DOI:** 10.1038/sj.bjc.6605280

**Published:** 2009-09-29

**Authors:** N M A White, M Mathews, G M Yousef, A Prizada, C Popadiuk, J J E Doré

**Affiliations:** 1 Division of BioMedical Sciences, Memorial University, St John's, Newfoundland, Canada; 2 Division of Community Health and Humanities, Memorial University, St John's, Newfoundland, Canada; 3 Department of Laboratory Medicine, The Keenan Research Centre, The Li Ka Shing Knowledge Institute, St Michael's Hospital, Toronto, Ontario, Canada; 4 Discipline of Laboratory Medicine, Memorial University, St John's, Newfoundland, Canada; 5 Discipline of Obstetrics and Gynecology, Health Sciences Centre, St John's, Newfoundland, Canada

**Keywords:** human kallikrein-related peptidase 6, human kallikrein-related peptidase 13, ovarian cancer, ovarian carcinogenesis, biomarkers

## Abstract

**Background:**

The human kallikrein-related peptidase family consists of 15 genes. Twelve of these genes are overexpressed in ovarian cancer and may represent potential markers for diagnosis, prognosis, and/or response to treatment. The aim of this study was to determine the prognostic significance of kallikrein-related peptidase 6 (*KLK6*) and kallikrein-related peptidase 13 (*KLK13*) in epithelial ovarian cancer by quantifying gene expression levels with tumour pathology and patient survival data.

**Methods:**

Total RNA was isolated from 106 patients diagnosed with primary ovarian cancer, as well as 8 normal ovary controls. Samples were analysed by quantitative real-time PCR for *KLK6* and *KLK13* expression. Correlation between kallikrein gene expression and clinical characteristics was evaluated with the *χ*^2^-test. Survival analysis was performed using Kaplan–Meier and Cox proportional hazards regression models.

**Results:**

Expression levels of both *KLK6* and *KLK13* mRNA were significantly increased in invasive cancers relative to normal ovaries (*P*=0.002 and 0.039 respectively). High *KLK6* and *KLK13* expression was an indicator of poor prognosis, with patients having a shorter recurrence-free survival (*P*=0.002 and 0.027 respectively). High *KLK6* expression was also significantly associated with lower overall survival (*P*=0.011). When subjected to multivariate analysis, patients with either high *KLK6* or *KLK13* were 3- and 2.2-fold, respectively, more likely to have a recurrence than patients with low kallikrein expression.

**Conclusion:**

These data show increased mRNA expression of *KLK6* and *KLK13* in ovarian cancer compared to normal ovarian tissues. High *KLK6* or *KLK13* expression in primary ovarian tumours can significantly predict prognosis in terms of recurrence-free survival and overall survival. In all, this study shows *KLK6* and *KLK13* as potential biomarkers and may be therapeutic targets for treatment of ovarian cancer.

Ovarian carcinoma, known as ‘the disease that whispers,’ is the most lethal of all the gynaecological malignancies. Annually, approximately 23 000 new cases and 14 000 deaths in the United States are due to ovarian cancer (Holschneider and Berek, 2000). This malignancy presents a great clinical challenge as it is often diagnosed in the late stages due to its anatomical location and relative asymptomatic occurrence (Holschneider and Berek, 2000). Approximately 75% of patients are diagnosed in late stage disease (stage III/IV) and have a 5-year survival rate of only 15–20%, compared to a 80–90% 5-year survival rate when diagnosed in the early stages (stage I/II; Schink, 1999). The disease is treatable and in most cases curable if diagnosed in the early stages.

Recently, a family of serine proteases has been identified on human chromosome 19q13 and named the human kallikrein-related peptidase family (KLK; Yousef and Diamandis, 2003). The family consists of 15 genes, of which 12 *(KLK2*, *KLK3*, *KLK4*, *KLK5*, *KLK6*, *KLK7*, *KLK8*, *KLK10*, *KLK11*, *KLK13*, *KLK14*, and *KLK15*) appear to be overexpressed in ovarian cancer. As been shown for prostate-specific antigen (PSA/*KLK3*) in prostate cancer, these may represent potential novel biomarkers for ovarian carcinoma (Borgono and Diamandis, 2004). This study focuses on two members of the kallikrein-related peptidase family, *KLK6* and *KLK13*.

Kallikrein-related peptidase 6 (*KLK6*) was initially identified by three different groups who named it protease M in breast cancer (Anisowicz *et al*, 1996), zyme in Alzheimer's disease (Little *et al*, 1997), and neurosin in colon adenocarcinoma (Yamashiro *et al*, 1997). *KLK6* is reported to have differential expression in ovarian, breast, uterine, and colon cancers (Anisowicz *et al*, 1996; Tanimoto *et al*, 2001; Hoffman *et al*, 2002; Ogawa *et al*, 2005; Santin *et al*, 2005). *KLK6* is overexpressed at both the gene and protein levels in ovarian cancer and has been associated with poor patient prognosis (Diamandis *et al*, 2000a; Tanimoto *et al*, 2001; Kountourakis *et al*, 2008). Recently, *KLK6* has been implicated in the loss of cell–cell contact and promotion of cell proliferation, migration, and invasion in keratinocytes (Klucky *et al*, 2007). With the involvement in these biological functions, the overexpression of *KLK6* in ovarian cancer suggests it may be involved in promoting cancer invasion and metastasis. *In vitro* assays have shown that recombinant KLK6 proteins are capable of extracellular matrix (ECM) protein digestion and neutralising KLK6 antibodies can decrease the rate of migration of ovarian cancer cell lines, further supporting this hypothesis (Ghosh *et al*, 2004).

Kallikrein-related peptidase 13 (KLK13) was first identified as downregulated in breast cancer tissues and cell lines (Yousef *et al*, 2000). However, 50% of malignant ovarian tissues had increased KLK13 expression relative to nearly undetectable levels in normal or benign tissue (Kapadia *et al*, 2003). In addition, Scorilas *et al* (2004) found high levels of KLK13 in early stage cancers and consequently associated high KLK13 with a better prognosis. Similar to KLK6, KLK13 can degrade major components of the ECM and when treated with an anti-KLK13 antibody, an ovarian cancer cell line showed decreased migratory capacity (Kapadia *et al*, 2004). On the basis of these previously observed *KLK6* and *KLK13* effects on ovarian cancer cells, the aim of this study was to evaluate the prognostic significance of *KLK6* and *KLK13* in epithelial ovarian cancer by quantifying gene expression levels and correlating them with clinical variables and patient survival data.

## Materials and methods

### Ovarian cancer samples

The study analysed formalin-fixed, paraffin-embedded ovarian tissues from 106 cases of sporadic ovarian carcinoma diagnosed in the province of Newfoundland and Labrador, Canada between 1983 and 2002. Eight normal ovary samples were also obtained for comparison. Tissues were collected from pathology archives and selected based on hematoxylin and eosin stains reviewed by a pathologist. Clinical staging was performed using the standard International Federation of Gynecology and Obstetrics staging, with tumours graded as borderline, well differentiated (grade I), moderately differentiated (grade II), or poorly differentiated (grade III). Clinical history was obtained by review of patients’ medical records in accordance with Memorial University's human investigation committee protocol.

Medical treatment of patients consisted of a total abdominal hysterectomy, bilateral salpingo-oophorectomy, omentectomy, and tumour staging. When cancer was not in stage 1A grade 1 or borderline, patients went on to receive chemotherapy. As the scope of this study spans from 1983 to 2002, chemotherapy regimes changed over this time. Before 1995/96 patients received cisplatin and cyclophosphamide, given for 6–9 cycles, at the discretion of the physician. After 1995/96 with the introduction of taxanes, treatments involved the combining of taxol with either cisplatin or carboplatin. A small number of patients with late stage (3C and 4) disease before 1998 were also treated with chemotherapy as a neoadjuvant pre-surgery.

### Immunohistochemistry

Sections were cut 4 *μ*m thick and dried on glass slides overnight. Sections were deparaffinised in xylene and re-hydrated through decreasing graded alcohols. Antigen retrieval was performed using a pressure cooker for 10 min in citrate buffer (pH 6.0). Slides were incubated overnight in primary antibody, washed twice with phosphate-buffered saline (pH 7.4) followed by either of two detection methods. Detection of KLK6 immunocomplex was carried out using Envision (Dako, Mississauga, Ontario, Canada), whereas localisation of KLK13 immunocomplex was performed with LSAB+, Link, and streptavidin reagents (Dako). Immune complexes were visualised by incubating with diaminobenzidine and sections were counterstained with hematoxylin. Immunolocalisation of KLK6 was carried out using a rabbit polyclonal antibody (Diamandis *et al*, 2000b), whereas KLK13 localisation was carried out using a mouse monoclonal antibody, clone 33.1 (Kapadia *et al*, 2003).

### Quantitative real-time PCR

Five 10 *μ*M sections were cut from paraffin-embedded tissues for nucleic acid isolation. Total RNA was extracted using the High Pure RNA Paraffin kit (Roche, Indianapolis, IN, USA) according to the manufacturer's protocol. Total RNA concentration was determined spectrophotometrically at 260 nM and samples were stored at −80°C. The presence of high molecular weight total RNA was determined using a NanoDrop 1000 Spectrophotometer (NanoDrop Technologies Inc., Wilmington, DE, USA) and ethidium bromide staining of samples using formaldehyde gel electrophoresis. cDNA was synthesised using 2 *μ*g total RNA and Superscript First-Strand Synthesis System for RT–PCR (Invitrogen, Carlsbad, CA, USA) with random hexamers to ensure representation of all mRNA independent of polyadenylated tail.

Quantitative real-time polymerase chain reaction (qRT-PCR) was performed using the ABI Prism 7000 (Applied Biosystems, Foster City, CA, USA). Primer/probe sets were purchased as pre-made TaqMan Assays on Demand for *KLK13*, *KLK6*, and glyceraldehyde 3-phosphate dehydrogenase (*GAPDH*; described in Supplementary Table 1). Thermal cycling conditions were according to the manufacture's protocol and all reactions were performed in triplicate. Relative quantification was defined as the amount of the specific mRNA normalised to a normal ovary as determined using the comparative cycle threshold (*C*_T_) method. The relative target gene expression was defined as 2^−ΔΔCT^, where ΔΔ*C*_T_=Δ*C*_T normal ovary_–Δ*C*_T ovarian cancer_. A normal ovarian sample having the median level of expression for a specific target gene was chosen as the calibrator sample and used to normalise expression of all other samples (i.e. target gene expression=1). Δ*C*_T_ is defined as *C*_T target_–*C*_T *GAPDH*_, where the target genes were *KLK13* and *KLK6*. Relative to the calibrator sample, target gene relative expression (RE) was classified as being low or high kallikrein expression. The expression level for determining high expressing samples was defined as one standard deviation above the mean value for all normal ovarian RE, for each specific target gene. Samples with an RE equal to or above this value were classified as high expression, whereas samples below the cut-off were classified as low expression. The cut-off for *KLK6* was determined as 5.211 RE and *KLK13* as 0.981 RE. Using a standard deviation above the normal mean RE of each target gene allowed us to utilise the inherent variation of each target gene expression to independently determine its cut-off value.

### Statistical analysis

All statistical analysis was performed with the SPSS statistical package for PC (version 13.0; SPSS Inc., Chicago, IL, USA). The relationships between *KLK6* and *KLK13* mRNA expression and patient clinical characteristics were analysed with a *χ*^2^-test. For survival analysis, two end points were examined; cancer recurrence (defined as either a local recurrence or metastasis) and death. These end points were used to calculate the recurrence-free survival (RFS) and overall survival (OS) respectively. Recurrence-free survival is defined as the time from first diagnosis to the time of first detected recurrence or metastasis. Overall survival is defined as the time from initial diagnosis to the time of death. Two survival models, the Kaplan–Meier and the Cox proportional hazard regression, were used for the analysis. The Kaplan–Meier model was used to examine survival between the patients expressing kallikreins at low or high levels, whereas significance was measured with the log-rank test. The Cox proportional hazard regression model, using both univariate and multivariate models, was used to determine the hazard ratio.

## Results

### Immunohistochemical localisation of KLK6 and KLK13 in ovarian carcinoma

**Figure 1:**
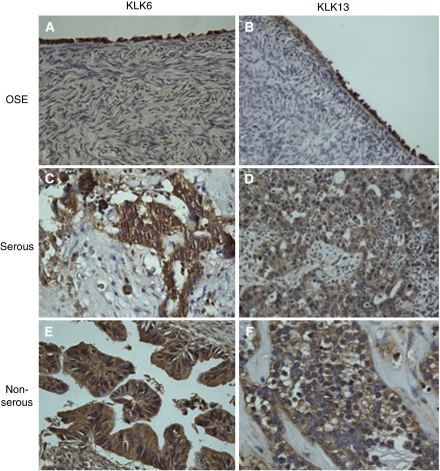
KLK6 and KLK13 expression in normal ovarian surface epithelium (OSE) and epithelial ovarian cancer. The normal OSE stains positive for both KLK6 (**A**) and KLK13 (**B**). Serous epithelial cancers express KLK6 (**C**) and KLK13 (**D**) in the cytoplasm. In a mucinous ovarian tumour, KLK6 shows strong expression (**E**). KLK13 is positively expressed in a clear cell ovarian tumour (**F**). All photomicrographs were taken at × 40 magnification.

Figure 1 illustrates the pattern of KLK6 and KLK13 localisation in ovarian surface epithelium (OSE) and epithelial ovarian carcinoma. Both KLK6 and KLK13 were localised in the cytoplasm of the normal ovarian surface epithelial (Figures 1A and B). Both KLK6 and KLK13 showed staining in all types of ovarian adenocarcinoma. Shown here is strong staining for both KLKs in serous adenocarcinoma (Figures 1C and D), with KLK6 shown expressed in a mucinous adenocarcinoma (Figure 1E) and KLK13 expression in a clear cell tumour (Figure 1F). In all cases, staining was most prominent in epithelial cells, whether they were normal surface epithelium or carcinoma. Immunohistological evaluation of protein levels resulted in no significant associations with clinical characteristics. The intensity of staining may not be only because of the variation of kallikrein expression in each pathological specimen, but also be contributed by the variations in fixing and embedding procedures used during the processing of tissues. To quantify the differences in expression more accurately, we extracted total RNA from ovarian samples and analysed by qRT-PCR, comparing KLK expression in ovarian tumours to normal samples.

### Association between Kallikrein gene expression and clinical variables

The relationship between *KLK6* and *KLK13* expression levels and clinical characteristics is summarised in Table 1. Patient ages range from 20 to 89 years with a mean age of 60 years. Compared to patients with low *KLK6*, a significantly larger proportion of patients with high *KLK6* had invasive cancer (*P*=0.002) and late stage cancers (*P*=0.001). Compared to patients with low *KLK13*, a larger proportion of patients with high *KLK13* had invasive cancer (*P*=0.039). Unlike *KLK6*, *KLK13* was not associated with clinical stage.

At diagnosis, 65% of all patients had serous ovarian cancer. In supplementary analysis, when we compared serous to non-serous ovarian cancers (mucinous, endometrioid, clear cell, and unknown), high *KLK6* expression was associated with serous carcinomas (*P*=0.001; data not shown). Interestingly, all endometrioid ovarian cancers had high *KLK13* expression relative to normal ovaries, but the small sample size was unable to provide sufficient power for a conclusive association.

### *KLK6* expression association with recurrence and survival

**Figure 2:**
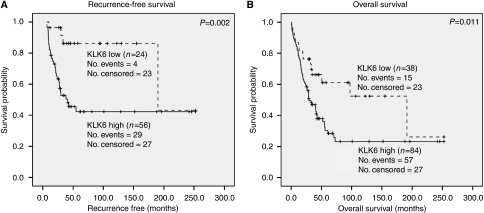
Kaplan–Meier survival curves showing the proportion of patients who are recurrence free (**A**) and alive (**B**) at the given time after diagnosis. Patients are stratified based on *KLK6* expression and compared by the log-rank test. Patients whose tumours are *KLK6* low are represented with the broken line, whereas patients whose tumours are *KLK6* high are represented by the solid line. *n*, number of patients.

Kaplan–Meier survival curves indicated patients with high *KLK6* expression were more likely to have a shorter RFS (*P*=0.002, Figure 2A) and OS (*P*=0.011, Figure 2B), when compared to patients with low *KLK6* expression. These data are further supported by the Cox regression analysis presented in Table 2. In univariate analysis, patients with high *KLK6* expression had a greater risk of recurrence (*P*=0.004) than patients with low *KLK6* expressing tumours. As expected, clinical stage (*P*<0.001), tumour grade (*P*=0.012), and histological type (*P*=0.024) were all significant predictors of recurrence. In the multivariate model (Table 3), high *KLK6* expression remains significant as a predictor of recurrence (*P*=0.040), indicating these patients are approximately three times more likely to have a recurrence than patients with low *KLK6* expression. Overall, late clinical stage (stage III/IV) was the strongest predictor of recurrence (*P*=0.001).

When OS is examined in a Cox univariate model (Table 2), high *KLK6* expression is significantly associated with a shorter OS (*P*=0.013). As expected, clinical stage (*P*<0.001), tumour grade (*P*<0.001), and histological type (*P*=0.037) are all associated with a shorter OS. Interestingly, patients 50 years of age or older, at the time of diagnosis, also had a significantly shorter OS (*P*=0.045) than patients under the age of 50. When these factors were included in a multivariate analysis (Table 3), clinical stage was the strongest predictor of OS, patients with late stage (stage III/IV) cancers had a four-fold increased likelihood of a shorter OS (*P*=0.001) than patients with early stage (Stage I/II) cancer. Other clinical characteristics lost their predictive significance of OS when subjected to multivariate analysis, including high *KLK6* expression (*P*=0.215).

### High *KLK13* expression in ovarian tumours is associated with poor prognosis

**Figure 3:**
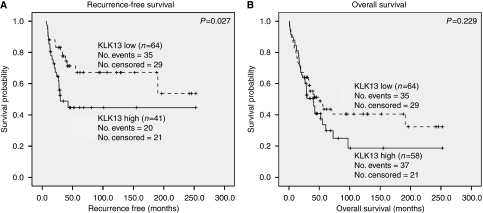
Kaplan–Meier survival curves showing the proportion of patients who are recurrence free (**A**) and alive (**B**) at the given time following diagnosis. Patients are stratified based on *KLK13* expression and compared by the log-rank test. Patients whose tumours are *KLK13* low are represented with the broken line, whereas patients whose tumours are *KLK13* high are represented by the solid line. *n*, number of patients.

When *KLK13* expression was analysed with the Kaplan–Meier model, patients with high *KLK13* expression had a shorter RFS than patients with low *KLK13* expression (*P*=0.027; Figure 3A). The strength of association between *KLK13* high expressing tumours and survival outcome is presented in a Cox regression model (Tables 3 and 4). Univariate analysis showed high *KLK13* as a significant predictor of recurrence (*P*=0.030), indicating a 2.2-fold increased probability of recurrence compared to low *KLK13* (Table 3). When *KLK13* expression was examined in a multivariate model, it retains the ability to significantly predict a shorter RFS (*P*=0.047; Table 4). Late clinical stage and serous type cancer also significantly predict a shorter RFS (*P*=0.001 and 0.024 respectively). When we examine *KLK13* expression along with other factors in a multivariate model, late clinical stage was the only significant predictor of OS (*P*<0.001).

## Discussion

Unlike other reproductive malignancies, such as prostate cancer, ovarian cancer lacks a biomarker that may be used for screening. Currently, CA125, the only marker used in ovarian cancer patients, is reliable only for monitoring response to treatment and disease recurrence. The identification of early biomarkers for ovarian cancer may lead to novel therapeutic applications and potential screening tests. This study was aimed at examining the expression of *KLK6* and *KLK13* in ovarian cancer to determine their diagnostic or prognostic value.

When we examined kallikrein protein expression by immunohistochemistry (Figure 1), we found that although there appeared to be increased KLK6 and KLK13 expression in ovarian cancer tumours relative to normal OSE, there were no significant associations to clinical variables or survival. These results are similar to previous studies that found increased expression of KLK6 in primary pancreatic ductal adenocarcinoma (Ruckert *et al*, 2008) and salivary gland tumours (Darling *et al*, 2006), but found no significant association with survival. Kallikreins 6 and 13 are expressed in normal epithelium (Petraki *et al*, 2001, 2003), including the OSE as we have shown here. Ovarian cancer is thought to arise from either OSE or OSE cells bordering inclusion cysts (Kaku *et al*, 2003), therefore it is expected that ovarian cancer cells would express a basal level of kallikrein expression. Given the semi-quantitative nature of immunohistochemistry and the fact that KLKs are secreted proteins, to see a significant difference in the cellular levels between normal OSE and ovarian cancer would require not only changes in KLK protein production, but also a significant change in the rate of kallikrein exocytosis.

With these limitations in quantifying protein expression, we used qRT-PCR to assess kallikrein mRNA expression in ovarian cancer tissues. High *KLK6* mRNA expression was associated with the presence of serous ovarian cancer and late stage disease. These results are similar to previous studies, which found increased *KLK6* expression in ovarian cancers when compared to normal ovarian tissue (Anisowicz *et al*, 1996; Tanimoto *et al*, 2001; Ni *et al*, 2004). Interestingly, previous studies have also associated high KLK6 serum levels with advanced ovarian cancer (stage III/IV) and serous tumour histology (Hoffman *et al*, 2002; Shan *et al*, 2007). Ovarian serous tumours of borderline and low grade are thought to arise from a step-wise progression from adenoma to borderline tumour to carcinoma through the *Ras-Raf* signalling pathway (Bell, 2005). Recently, *KLK6* expression and secretion has been shown to be Ras dependent in a colon carcinoma cell line (Henkhaus *et al*, 2008). Constitutively active mutant K-Ras resulted in enhanced colon cancer cell invasion through both laminin and Matrigel matrixes. Together, these data suggest that *Ras-Raf* mutations may increase the invasive potential of these borderline tumours through increased expression of KLK6. This may also hold true for advanced ovarian cancers, as we show high KLK6 expression is associated with later stage, more invasive, cancers. High-grade serous carcinomas have a notably different pathogenesis than low-grade serous tumours. High-grade serous tumours commonly have mutations in p53, BRCA 1, and/or BRCA2 (Christie and Oehler, 2006). To date there have be no reports indicating a relationship between KLK6 and p53 or BRCA genes. When we looked at only high- and low-grade serous tumours, we found no significant differences in KLK6 expression (*P*=0.498). Despite the apparent differences in the molecular oncogenesis of low- and high-grade serous tumours, their upregulation of KLK6 suggests a common pathway is activated in both types of tumours.

Our study is the first to report *KLK13* mRNA expression in normal ovary and ovarian cancer patients. The expression of *KLK13* mRNA in normal OSE was extremely low, whereas 55% ovarian cancers examined had high *KLK13* expression. These findings support Kapadia *et al* (2003), who found serum levels of KLK13 were below levels of detection in healthy individuals, yet 50% ovarian cancer patients were positive for KLK13 (Kapadia *et al*, 2003). Interestingly, 100% of our endometrioid cancer samples were high grade and expressed *KLK13* at a high level. Although our sample size is small (six cases), this expression pattern warrants further investigation. Low-grade endometrioid carcinomas have been suggested to arise from endometriosis or borderline endometrioid tumours (Obata *et al*, 1998), whereas high-grade endometrioid carcinomas have changes similar to high-grade serous carcinomas (Bell, 2005; Giordano *et al*, 2008; Press *et al*, 2008) suggesting endometrioid cancers may represent two separate malignancies. This characteristic expression pattern of KLK13 may represent not only a novel marker to distinguish between high- and low-grade endometrioid cancers, but a unique pathway in which KLK13 may be involved in ovarian carcinogenesis.

Since histological types of ovarian cancers present as a spectrum of pathologically and histologically different phenotypes and may represent cancers of different origins (Bell, 2005), the possibility that a panel of markers, rather than a single marker, may improve the sensitivity and specificity of detecting ovarian cancer at an early stage while it is treatable. Zheng *et al* (2007) found that a group of kallikrein-related peptidases, including KLK6 and KLK13, in multi-parametric combinations with other biomarkers and clinical variables can significantly predict prognosis and response to treatment in ovarian cancer patients. A similar model, including a number of kallikrein-related peptidases, has been proposed for non-small-cell lung carcinoma (Planque *et al*, 2008). This study confirms both KLK6 and KLK13 are overexpressed in ovarian cancer and are useful predictors of poor prognosis in ovarian cancer patients. This study is the first to report upregulation of *KLK13* mRNA in ovarian cancer patients and indicated that *KLK13* may represent a specific marker for endometrioid carcinoma. These findings support the potential role as kallikrein-related peptidases 6 and 13 as novel ovarian cancer biomarkers and may, in the future, offer targets for therapeutic applications.

## Figures and Tables

**Table 1:** Association between *KLK6* and *KLK13* mRNA expression with clinicopathological data for 106 ovarian carcinoma patients and 8 normal ovaries

		**No. of patients (%)**			**No. of patients (%)**		
**Variable**	***n***	***KLK6* low**	***KLK6* high**	***P*-value**	***KLK13* low**	***KLK13* high**	***P*-value**
*Age*							
<50	24	8 (33.3)	16 (19.5)	0.155	9 (18.8)	15 (25.9)	0.384
⩾50	82	16 (66.7)	66 (80.5)		39 (81.3)	43 (74.1)	
*Status*							
Normal	8	6 (20.0)	2 (2.4)	0.002	7 (12.7)	1 (1.7)	0.039
Borderline	6	3 (10.0)	3 (3.6)		4 (7.3)	2 (3.4)	
Invasive	100	21 (70.0)	79 (94.0)		44 (80.0)	56 (94.9)	
*Histological type*							
Serous	69	10 (41.7)	59 (72.0)	0.073	33 (68.8)	36 (62.1)	0.082
Mucinous	15	6 (25.0)	9 (11.0)		5 (10.4)	10 (17.2)	
Endometroid	6	3 (12.5)	3 (3.7)		0	6 (10.3)	
Clear cell	4	1 (4.2)	3 (3.7)		2 (4.2)	2 (3.4)	
Unknown	12	4 (16.7)	8 (9.8)		8 (16.7)	4 (6.9)	
*Clinical stage*							
Early (I/II)	32	14 (25.0)	18 (22.0)	0.001	16 (33.3)	16 (27.6)	0.521
Late (III/IV)	74	10 (14.7)	64 (78.0)		32 (66.7)	42 (72.4)	
*Tumour grade*							
GB/G1	21	6 (25.0)	15 (18.3)	0.468	12 (25.0)	9 (15.5)	0.223
G2/G3	85	18 (75.0)	67 (81.7)		36 (75.0)	49 (84.5)	

**Table 2:** Univariate Cox regression analysis of *KLK6*, *KLK13*, and other clinicopathological variables

	**RFS**			**OS**		
**Variable**	**HR**	**95% CI**	***P*-value**	**HR**	**95% CI**	***P*-value**
*Univariate analysis*						
*KLK6*						
Low	1.00	—	—	1.00	—	—
High	4.59	1.61–13.08	0.004	2.06	1.16–3.63	0.013
*KLK13*						
Low	1.00	—	—	1.00	—	—
High	2.19	1.08–4.46	0.030	1.33	0.83–2.19	0.231
Clinical stage^a^ (ordinal)	11.89	3.61–39.15	<0.001	5.91	2.82–12.35	<0.001
Tumour grade^b^ (ordinal)	3.42	1.31–8.94	0.012	3.83	1.82–8.07	<0.001
Histological type^c^ (ordinal)	2.31	1.12–4.78	0.024	1.67	1.03–2.71	0.037
Age^d^ (ordinal)	2.76	0.97–7.87	0.057	1.98	1.02–3.87	0.045

Abbreviations: 95% CI=95% confidence interval; HR=hazard ratio; OS=overall survival; RFS=recurrence-free survival.

a Clinical stage: late *vs* early stage.

b Tumour grade: poor *vs* well differentiated.

c Histological type : serous *vs* non-serous.

d Age: ⩾50 *vs* <50.

**Table 3:** Multivariate Cox regression analysis of *KLK6* expression

	**RFS**			**OS**		
**Variable**	**HR**	**95% CI**	***P*-value**	**HR**	**95% CI**	***P*-value**
*Multivariate analysis*						
*KLK6*						
Low	1.00	—	—	1.00	—	—
High	3.03	1.05–8.74	0.040	1.45	0.81–2.60	0.215
Clinical stage^a^ (ordinal)	8.57	2.45–30.05	0.001	3.73	1.70–8.18	0.001
Tumour grade^b^ (ordinal)	1.48	0.52–4.16	0.457	2.08	0.94–4.63	0.073
Histological type^c^ (ordinal)	1.92	0.91–4.04	0.087	1.25	0.77–2.04	0.367
Age^d^ (ordinal)	2.19	0.72–6.67	0.167	1.47	0.74–2.94	0.271

Abbreviations: 95% CI=95% confidence interval; HR=hazard ratio; OS=overall survival; RFS=recurrence-free survival.

a Clinical stage: late *vs* early stage.

b Tumour grade: poor *vs* well differentiated.

c Histological type : serous *vs* non-serous.

d Age: ⩾50 *vs* <50.

**Table 4:** Multivariate Cox regression analysis of *KLK13* expression

	**RFS**			**OS**		
**Variable**	**HR**	**95% CI**	***P*-value**	**HR**	**95% CI**	***P*-value**
*Multivariate analysis*						
*KLK13*						
Low	1.00	—	—	1.00	—	—
High	2.20	1.01–4.78	0.047	1.00	0.61–1.61	0.988
Clinical stage^a^ (ordinal)	9.08	2.61–31.65	0.001	4.00	1.84–8.70	<0.001
Tumour grade^b^ (ordinal)	1.22	0.42–3.55	0.717	2.14	0.95–4.81	0.065
Histological type^c^ (ordinal)	2.43	1.13–5.24	0.024	1.29	0.79–2.10	0.308
Age^d^ (ordinal)	2.49	0.82–7.54	0.108	1.46	0.73–2.91	0.286

Abbreviations: 95% CI=95% confidence interval; HR=hazard ratio; OS=overall survival; RFS=recurrence-free survival.

a Clinical stage: late *vs* early stage.

b Tumour grade: poor *vs* well differentiated.

c Histological type : serous *vs* non-serous.

d Age: ⩾50 *vs* <50.
