# Supplementary material for: KLK6 and KLK13 predict tumor recurrence in epithelial ovarian carcinoma
Source: Br J Cancer. 2009 Sep 29;101(7):1107–13. doi: 10.1038/sj.bjc.6605280 (PMC2768090; doi:10.1038/sj.bjc.6605280)
Supplement: Supplementary Table 1 [file 6605280x1.pdf]

Supplementary Table 1. TaqMan Probes and characteristics used for qRT- PCR

Probe	Target	Accession Number	Transcript Variants Detected	mRNAs Detected	Amplicon Length	Spanned Exons
Hs00160519_m1	KLK6[1]	NM_002774.3<br>NM_001012964.1<br>NM_001012965.1	Transcript Variant A<br>Transcript Variant B<br>Transcript Variant C	13	119bp	2-3
Hs01087307_m1	KLK13[2]	NM_015596.1	Splice Variant 1<br>Splice Variant 2<br>Splice Variant 3<br>Splice Variant 4<br>Splice Variant 5<br>Splice Variant 6<br>Splice Variant 7<br>Splice Variant 8	10	82bp	1-2
Hs99999905_m1	GAPDH[3]	NM_002046.3	N/A	121	122bp	2-3

1. Human kallikrein related peptidase 6
2. Human kallikrein related peptidase 13
3. Glyceraldehyde 3-phosphate dehydrogenase
